# Managing patients with dengue fever during an epidemic: the importance of a hydration tent and of a multidisciplinary approach

**DOI:** 10.1186/1756-0500-4-335

**Published:** 2011-09-08

**Authors:** Alexandre R Marra, Gustavo Faissol Janot de Matos, Renata Donato Janeri, Patricia Sousa Machado, Claudio Schvartsman, Oscar Fernando Pavão dos Santos

**Affiliations:** 1 Intensive Care Unit, Hospital Israelita Albert Einstein, São Paulo, Brazil; 2 Emergency Department, Hospital Israelita Albert Einstein, São Paulo, Brazil; 3 Pediatric Department, Hospital Israelita Albert Einstein, São Paulo, Brazil

## Abstract

**Background:**

Dengue fever is one of the most common tropical diseases worldwide. Early detection of the disease, followed by intravenous fluid therapy in patients with dengue hemorrhagic fever (DHF) or with warning signs of dengue has a major impact on the prognosis. The purpose of this study is to describe the care provided in a hydration tent, including early detection, treatment, and serial follow-up of patients with dengue fever.

**Findings:**

The analysis included all patients treated in the hydration tent from April 8 to May 9, 2008. The tent was set up inside the premises of the 2^nd ^Military Firemen Group, located in Meier, a neighborhood in Rio de Janeiro, Brazil. The case form data were stored in a computerized database for subsequent assessment. Patients were referred to the tent from primary care units and from secondary city and state hospitals. The routine procedure consisted of an initial screening including vital signs (temperature, blood pressure, heart rate, and respiratory rate), tourniquet test and blood sampling for complete blood count. Over a 31-day period, 3,393 case recordings were seen at the hydration tent. The mean was 109 patients per day. A total of 2,102 initial visits and 1,291 return visits were conducted. Of the patients who returned to the hydration tent for reevaluation, 850 returned once, 230 returned twice, 114 returned three times, and 97 returned four times or more. Overall, 93 (5.3%) patients with DHF seen at the tent were transferred to a tertiary hospital. There were no deaths among these patients.

**Discussion:**

As the epidemics were already widespread and there were no technical conditions for routine serology, all cases of suspected dengue fever were treated as such. Implementing hydration tents decrease the number of dengue fever hospitalizations.

## Background

Dengue fever is one of the most common tropical diseases worldwide and is caused by a *Flavivirus *transmitted to humans by infected *Aedes aegypti *mosquitoes [1]. The natural habitat of this disease comprises tropical and subtropical regions with warm and humid climates. The mosquito will reproduce in small water collections such as in flower vases, uncovered water storage vessels, used automobile tires and any place where clean water might be trapped; therefore, its control is very difficult and highly dependent on the local population's sanitary habits [2]. Due to its geographic characteristics, the city of Rio de Janeiro has long suffered dengue fever outbreaks and epidemics.

Early detection of the disease, followed by intravenous fluid therapy in patients with dengue hemorrhagic fever (DHF) or with complicated dengue fever has a major impact on the prognosis [1,3,4].

In 2008, in the city of Rio de Janeiro, Brazil, 232 deaths from dengue fever were officially recorded [5]. This figure exceeded the one reported during the previous epidemic of 2001, when 91 people died of the disease. In the whole State of Rio de Janeiro, 143,350 cases were registered in the period from January 1^st ^to May 30^th ^2008. In 2002, 288,245 cases were recorded. Children under 15 years of age accounted for 46% of all deaths in 2002 [6,7].

Aware of this alarming situation, the Albert Einstein Hospital in São Paulo, Brazil, volunteered to provide support for healthcare authorities in Rio de Janeiro by sending a multidisciplinary team and medical supplies. The objective was to organize and supply a hydration tent to the victims of dengue fever. This paper contains a description of the care provided in a hydration tent, including early detection, treatment and transfer of the severe cases to the hospitals.

## Materials and methods

The analysis included for all patients treated in the hydration tent from April 8 to May 9, 2008. The tent was set up inside the premises of the 2^nd ^Military Firemen Group, located in Meier, a neighborhood of Rio de Janeiro, Brazil. The case form data were stored in a computerized (an electronic) database for subsequent assessment.

As the epidemics were already widespread and (there were no technical conditions for) routine serology could not be performed given the technical circumstances, all cases of suspected dengue fever were treated as such. This fact might have influenced the total number of cases recorded. However, the Rio de Janeiro State healthcare agency recommended any dengue fever case with suggestive symptoms be considered as such. In this context, it is not feasible to perform universal serology testing for dengue fever in the affected population.

### Characteristics of the hydration tent and the multidisciplinary team

The tent covered an area of 1,400 sq. ft. and included a waiting room with 20 seats, a medical office with two tables and one stretcher, a small cool room for blood count tests and a room for intravenous fluid replacement with 30 armchairs.

The hydration tent was open and operational around the clock. It was equipped with supplies for venipuncture, intravenous solutions, an automated blood cell analyzer (Coulter T-890, Diamond Diagnostics, US) that released results within 40 seconds and symptomatic medication (metamizole, paracetamol, metoclopramide, and dimenhydrate). The multidisciplinary team consisted of 197 professionals from the Albert Einstein Hospital staff, in São Paulo, Brazil, including 81 nursing assistants, 48 nurses, 51 medical doctors, 8 lab technicians, and 9 administrative support employees. In each 12-hour shift, a team of 4 medical doctors (2 general practitioners [GPs] and 2 pediatricians), 3 nurses, 6 nursing assistants, 2 administrative clerks and 1 lab technician were on duty.

### Patient care flow and rehydration therapy

Patients were referred to the tent from primary care units and from secondary city and State hospitals. The routine procedure consisted of an initial screening including vital signs (temperature, blood pressure, heart rate, and respiratory rate), tourniquet test and blood sampling for complete blood count (CBC). Once screened, the patients were seen either by a pediatrician or by a GP and evaluated through a detailed medical history and physical examination, besides an analysis of the CBC report. If the clinical diagnosis of dengue fever was confirmed, the patient was assigned to one of three groups: A - oral rehydration therapy (ORT), B - intravenous rehydration therapy (IVT), or C - discharge with recommendations.

The dehydration status was assessed during the physical examination by the doctor or through the hematocrit, performed together with the CBC. We complied with the WHO case definition of dengue hemorrhagic fever (DHF) [3].

The decision to start ORT or IVT was taken by the physician, based on clinical and laboratory (CBC) criteria [3]. ORT consisted of encouraging fluid ingestion, under supervision, either at the water fountains installed in the tent or by volunteers who constantly offered the patients filtered water. In addition, the patients received instructions about fluid ingestion at home. IVT consisted of the intravenous administration of normal saline or 5% dextrose with electrolytes. Some of these patients had nausea or vomiting and we prescribed intravenous antiemetics.

After ORT or IVT, patients were reassessed and a new CBC was performed to check the effectiveness of the rehydration measures. If there were no signs of clinical and laboratorial improvement, patients received another course of intravenous fluid therapy. They were then reassessed and, if necessary, transferred to a tertiary care hospital. Patients showing improvement were discharged and instructed to return if they experienced symptoms of disease progression, such as dehydration, persistent vomiting or diarrhea, bleeding events or signs of hemodynamic instability. In addition, they received an ID card with data on their hematocrit, platelet count and vital signs. The patients were instructed to bring along this card in case they returned to the tent for further assessment. Supportive serology tests (IgM antibody test on a late acute or convalescent-phase serum specimen) were performed only for in-patients with DHF [1]. None of the cases (except DHF) were confirmed by specific laboratory tests. Patients up to 15 years of age were considered pediatric patients.

The DHF cases were hospitalized. These DHF cases could be identified either at the first visit or in the course of the disease. When such a case (DHF) was identified, our team immediately triggered (mobilized) a medical call center asking for the patient's hospitalization in a public hospital in Rio de Janeiro city. The data on the outcome of these cases (hospital discharge or death) were received from the Rio de Janeiro State healthcare agency.

## Results

Over a 31-day period, 3,393 case recordings were seen at the hydration tent in Meier, Rio de Janeiro. The mean was 109 patients per day, with a peak of 232 visits on April 14, 2008.

The total number of case recordings studied included 2,594 (76.5%) adults and 799 (23.5%) pediatric patients. A total of 2,102 initial visits and 1,291 return visits were conducted. In the 2,102 initial visits, 1,739 patients had clinical symptoms that were suggestive of dengue fever; 363 patients did not present clinical symptoms suggestive of dengue fever and had other differential diagnoses. Of the 1,739 patients with clinical symptoms of dengue fever, 1,320 were adults (75.9%) and 419 were children (24.1%). Of the case recordings that returned to the hydration tent for reevaluation (n = 1,291), 850 returned once, 230 returned twice, 114 returned three times and 97 returned four times or more (to a maximum of 8 times). A total of 363 patients (with 407 visits) did not have clinical symptoms that were suggestive of dengue fever and the most common differential diagnoses detected at the Tent's Triage are depicted in Table 1. In our analysis we have considered only dengue fever cases.

**Table 1:** Most common differential diagnosis detected at Tent's Triage (N = 363)

Symptoms	%
Upper Airway Infection	43
Gastroenterocolitis	25
Community Acquired Pneumonia	6
Bacterial Amigdalitis	6
Sinusitis	3
Migraine	3
Appendicitis	2
Rubeolla	1
Hypoglicemia	1
Other	10

The most frequent clinical symptoms suggestive of dengue fever reported by adults at the first visit (n = 1,320) were: headache (77%), fever (70%), myalgia (69%), retro-orbital pain (38%), abdominal pain (32%), nausea (32%), vomiting (28%), diarrhea (23%), rash (8%), and dyspnea (6%). In children (n = 419) the most common symptoms were: fever (85%), headache (59%), vomiting (42%), myalgia (38%), abdominal pain (33%), diarrhea (19%), retro-orbital pain (18%), nausea (16%), rash (16%), and dyspnea (3%). Only 10% of all of the cases (n = 1,739) had a positive tourniquet test.

In adults (n = 1,320), laboratory test results showed mean values of hematocrit, platelet count and leukocyte count at the initial visit of 41.3 ± 8.9%, 176.0 ± 91.3 (10^3^/mm^3^), and 5.3 ± 3.8 (10^3^/mm^3^) respectively. In children (n = 419), the mean values of hematocrit, platelet count and leukocyte count at the time of the initial visit were 39.3 ± 4.1%, 211.0 ± 92.9 (10^3^/mm^3^), and 6.4 ± 4.5 (10^3^/mm^3^), respectively.

As to what concerns treatment modality, 824 case recordings (24.3%) received IVT. Of this total, 230 case recordings (28%) required a reevaluation after the initial intravenous fluid therapy and had a second CBC performed during the same visit. IVT was used in 117 children (27.9% of the pediatric patients, n = 419) and in 365 adults (27.7% of the adult patients, n = 1320).

In the adult population (n = 1,320), 172 patients had platelet counts lower than 100,000/mm^3 ^(13.0%) and 86 also showed signs of hemoconcentration (6.5%). In the pediatric population (n = 419), 56 patients had platelet counts lower than 100,000/mm^3 ^(13.4%), and 55 also showed signs of hemoconcentration (13.1%).

Overall, 93 (5.3%) patients with DHF seen at the tent were transferred to a tertiary hospital. Of these, 78 were adults (83.9%) and 15 were children (16.1%).

Despite the need for hospitalization in 5% of the cases due to the severity of the disease, there were no deaths among these patients. Moreover, our team avoided potential hospitalizations in dengue fever patients. This suggests that the initial therapeutic approach was effective. CBC reports were available for all the patients seen at the hydration tent within 40 seconds, which allowed immediate therapeutic decisions to be made based on the presence or absence of hemoconcentration and thrombocytopenia.

## Discussion

During the first half of 2008, the number of notified cases of dengue fever was overwhelming, reaching a level similar to that of the 2002 epidemic, the worst in the past 20 years. From January to July 2008, more than 120,000 cases were recorded at the Rio de Janeiro State healthcare agency, while 150,321 cases had been notified over the whole year 2002 [6,7]. These data reflected an extremely serious public health issue in Rio de Janeiro [8]. The massive demand for medical assistance from a public system that already operated at its maximum capacity resulted in long waiting lines, restricted access to healthcare services and a shortage of hospital beds, both in public and private sectors. This ultimately led to chaos in the city [8,9].

Patients who could have been diagnosed and treated at an earlier stage, using low-complexity diagnostic and therapeutic tools, progressed to more severe forms of the disease [10]. With the increasing number of cases, the shortage of available facilities and healthcare professionals soon became critical. In this context, some emergency measures, such as the implementation of hydration tents, proved to be a key factor in epidemic control. In total, 17 hydration tents were deployed in the State to receive patients referred by primary care units and emergency hospitals with symptoms suggestive of dengue fever. This was one of the reasons for offering help to the city of Rio de Janeiro, and sending a multidisciplinary team with a hydration tent in order to lower the disease morbidity, the number of hospitalizations and if possible, the mortality rates. If any major complication was detected, the patient was transferred to a tertiary hospital. Over the study period, there were no patient deaths among those treated at the Meier hydration tent. The multidisciplinary team consisted for professionals from various sectors of our hospital (intensive care, emergency, pediatrics, surgery, etc.). All engage in volunteer work providing care to all patients who visited the tent of hydration.

Significantly, 25% of all the patients seen at our hydration tent could be treated with intravenous fluid therapy on-site, thus avoiding unnecessary referral to overloaded hospitals.

A major contribution of this approach was to demonstrate that simple, low-cost measures were effective in the care of patients with dengue fever during the epidemics in Rio de Janeiro, and had the positive impact of possibly reducing not only patient mortality but also to avoid the hospitalization of dengue fever patients in the Rio de Janeiro crowded hospitals, which we believe was our most valuable contribution.

The kind of help provided reduced the waiting lines in the surrounding hospitals, decentralized services and allowed earlier diagnosis and treatment of the more severe cases. Another very positive result of this effort was the significant growth in knowledge and experience on the part of the multidisciplinary team. As they saw an increasing number of patients, the healthcare team felt more confident to make decisions and provide better patient care, thus avoiding unnecessary hospitalizations.

The clinical and laboratory data obtained were similar to those reported in the literature [1,11], and underline the relevance and potential magnitude of this disease when it is not immediately controlled. While dengue fever is, in theory, a low-complexity and easily manageable disease, it has potentially devastating consequences when there is a favorable environment for the fast proliferation of the mosquito, as it happens to be the case in Rio de Janeiro [8].

Several studies suggest different dengue vector strategies in urban settings [12,13] and try to achieve dengue control in many parts of the world [14,15]. Focus is also on the discussion about taking care of in-hospital dengue fever patients [16,17]. However, we did not find any similar study providing solutions for taking care of dengue fever patients outside hospitals.

Therefore, it is important to emphasize that the efforts made to provide care for patients with dengue fever during epidemics represent a major contribution to prevent further complications, including death. However, if there is no commitment to primary prevention, there will always be a need to treat the consequences of the disease instead of attacking its cause. A preventive attitude needs to be adopted not only by government bodies but also by the general population, who must improve their sanitary and hygienic habits in the near future.

### Limitations

Since all the patients that were followed at the Tent were referred from other hospitals or ambulatory clinics, they might have been previously medicated for symptoms such as pain, fever or nausea for example. Therefore, at the Tent's Triage, some patients may not have informed correctly about all the symptoms they might be feeling on that occasion (at the Tent's Triage). So the percentage of symptoms could have been underestimated.

## Conclusions

Although a manageable disease, dengue fever still represents a major challenge for public health care services in tropical countries with a favorable environment for the *Aedes aegypti*. Primary prevention measures produce a great impact on the control of epidemics such as the one observed in 2008, and must be urgently improved. In disaster situations, implementing hydration tents might change the fatal outcome of the more severe cases. The multidisciplinary team approach has proven to be essential for the effective management of the hydration tent.

## Competing interests

The authors declare that they have no competing interests.

## Authors' contributions

AM, GFJM, RJ and PSM participated in the data collected and data analysis. CS and OFPS participated in the design and cordination and helped to draft the manuscript.

All authors read and approved the final manuscript.
